# Phrenic Nerve Sonography Alterations in Patients with ALS: Insight with Clinical and Neurophysiological Findings

**DOI:** 10.3390/jcm13216302

**Published:** 2024-10-22

**Authors:** Ovidijus Laucius, Justinas Drūteika, Renata Balnytė, Jolita Palačionytė, Miglė Ališauskienė, Kęstutis Petrikonis, Antanas Vaitkus

**Affiliations:** Department of Neurology, Medical Academy, Lithuanian University of Health Sciences, 44307 Kaunas, Lithuania; justinas.druteika@lsmu.lt (J.D.); renata.balnyte@lsmu.lt (R.B.); jolita.palacionyte@lsmu.lt (J.P.); migle.alisauskiene@lsmu.lt (M.A.); kestutis.petrikonis@lsmu.lt (K.P.); antanas.vaitkus@lsmu.lt (A.V.)

**Keywords:** phrenic nerve, sonography, ultrasound, amyotrophic lateral sclerosis (ALS)

## Abstract

**Background:** Amyotrophic lateral sclerosis (ALS) is a progressive neurodegenerative disorder, and although the diagnosis is primarily based on clinical criteria, ENMG, as the “gold standard”, does not always show detectable changes. Therefore, our study suggests that alterations in echogenicity and heterogeneity of the phrenic nerve (PN) may serve as potential additional diagnostic tools for ALS. **Methods:** Our study included 32 patients in the ALS group and 64 individuals in the control group. Each participant underwent an interview and completed questionnaires to collect clinical and demographic data, including age, gender, height, body mass index (BMI), hip and waist circumference, duration of illness, ALS-FRS-R score, comorbidities, and medication use. Ultrasound examinations of the PN were performed by two authors using a high-resolution “Philips EPIQ 7” ultrasound machine equipped with a linear 4–18 MHz transducer. The ALS group participants underwent PN sonography and conduction examinations, arterial blood gas (ABG) analysis, respiratory function tests (RFT), and electroneuromyography (ENMG). **Results:** The study demonstrated that the phrenic nerve is significantly smaller on both sides in patients with ALS compared to the control group (*p* < 0.01). Changes in the homogeneity and echogenicity of the PN were also observed on both sides. On the right side, 43.8% of the nerves showed heterogeneity, 40.6% were isoechoic, and 21.9% were hyperechoic. On the left side, 59.4% of the nerves exhibited heterogeneity, 34.4% were isoechoic, and 28.1% were hyperechoic. Moreover, sonography on both sides showed significant correlation with ALS-FRS-R, COMPASS-31, and ENMG results. **Conclusions:** Our study highlights the importance of phrenic nerve ultrasound as a promising supplementary diagnostic tool for ALS. The significant differences in phrenic nerve size, echogenicity, and homogeneity between patients with ALS and the control group demonstrate that ultrasound imaging can detect morphological changes in the phrenic nerve. Incorporating phrenic nerve ultrasound into routine diagnostic protocols could improve early detection, enhance disease monitoring, and offer a more comprehensive understanding of the neurodegenerative processes in ALS.

## 1. Introduction

In our previous study [1], we hypothesized that the reduction of PN detectable via ultrasound could be one of the first signs of ALS, potentially offering valuable insights for early diagnosis and prognosis. In order to strengthen the diagnostic value of PN ultrasound, the current study aims to compare sonographic changes with functional measurements of nerve conduction and respiratory function.

Amyotrophic lateral sclerosis (ALS) is a progressive neurodegenerative disorder characterized by the degeneration of upper and lower motor neurons leading to muscle weakness, severe disability, and ultimately a fatal outcome [2,3,4]. The etiology of ALS remains largely unknown in most cases. Sporadic instances account for approximately 90–95% of all cases, whereas genetic factors play a role in 5–10% of occurrences [5,6]. The prevalence of ALS varies geographically. Regional incidence rates vary from 0.6 to 3.8 per 100,000 individuals, with a global incidence of approximately 1.6 to 1.76 per 100,000 population [6,7,8,9]. In Asian countries, incidence rates range from 0.7 to 2.2 cases per 100,000 population, while in more developed regions such as Europe and North America, rates are higher, ranging from 1.5 to 3.8 per 100,000 population [2,4,6,9,10,11,12]. Data on ALS within the Lithuanian population remain inconclusive; however, estimates suggest an incidence of 2 to 3.5 cases per 100,000 individuals. ALS can manifest at any age of life, with the risk increasing with each decade, particularly after the age of 40. Typically, the disease is most commonly diagnosed in older adults, with the median age at diagnosis between 51 and 66 years [13,14,15,16]. Males are slightly more affected than females until the age of 65 [6], with a higher incidence observed in males across all age groups. The peak prevalence age for males is 85–89 years, whereas for females, it is 80–84 years. The age-standardized male-to-female ratio increased from 1.22 (1.19:1.24) in 1990 to 1.25 (1.23:1.28).

Various pathogenic factors of the disease lead to a wide range of clinical features and symptoms in ALS presentation [16,17]. The diagnosis of amyotrophic lateral sclerosis is made using the Gold Coast Criteria [18], which have been developed to provide a more streamlined and standardized approach to diagnosing this condition. According to the Gold Coast criteria, ALS is diagnosed when there is evidence of both upper and lower motor neuron degeneration that is progressive and not explained by other conditions. Although the diagnosis of ALS is primarily based on clinical criteria and the monitoring of disease progression, various diagnostic tests can enhance the accuracy of patient assessment and reduce diagnostic errors [19].

The phrenic nerve, originating from the cervical spinal cord (C3–C5), provides the motor innervation necessary for diaphragmatic contraction, which is essential for generating negative intrathoracic pressure and facilitating lung inflation during inspiration [20]. Respiratory onset in ALS is uncommon, occurring in fewer than 5% of cases [21]. However, respiratory involvement typically develops during the course of the disease, and the progressive decline in phrenic nerve (PN) function leads to respiratory muscle weakness, paralysis, and ultimately, patient death [22,23]. Several studies have shown that ultrasound assessment of the PN and nerve conduction can help evaluate respiratory function and predict patient survival [5,6,24,25]. A more detailed investigation of the phrenic nerve is crucial due to its vital role in controlling the diaphragm, the primary muscle involved in respiration.

This study is the first of its kind conducted in Lithuania. We performed a comparative analysis of the phrenic nerve in terms of cross-sectional area, homogeneity, and echogenicity. These morphological parameters were evaluated alongside clinical indicators, blood sample analyses, nerve conduction studies, and assessments using the ALS functional Rating scale-revised (ALSFRS-R) and the composite autonomic symptom scale 31 (COMPASS-31). Significant attention is given to respiratory function, as its impairment occurs at various stages of the disease and is associated with a poorer prognosis [17,26]. This comprehensive approach allowed us to investigate the correlation between nerve ultrasound characteristics and various clinical and functional parameters in patients with ALS, with the aim of suggesting that it could serve as an additional tool in determining diagnosis.

## 2. Materials and Methods

### 2.1. Study Subjects

The study was carried out at the Department of Neurology of the Lithuanian University of Health Sciences. Participants in the ALS group were involved in the investigation between 2022 and 2023. Inclusion criteria for the study included patients diagnosed with amyotrophic lateral sclerosis according to the Gold Coast diagnostic criteria [18] aged over 18 years, with no other suspected diseases based on electrophysiological and imaging tests, no other neurodegenerative diseases identified, and no diagnosed neuromuscular junction disorders. Exclusion criteria involved patients younger than 18 years, lack of patient consent, pregnant women, and patients with electrophysiological or imaging findings suggesting another disease that could influence the study results.

The participants were divided into two groups: Group 1 consisted of patients with ALS (*n* = 32), who all met the “Gold Coast” criteria [18]. Group 2 served as the control group (*n* = 64). Each participant underwent an interview and filled out questionnaires to collect clinical and demographic data, including age, gender, height, body mass index (BMI), hip and waist circumference, duration of illness, ALS-FRS-R score, comorbid illness, and medication use. ALS group participants underwent a PN ultrasound and conduction examination, arterial blood gas (ABG), and respiratory functional tests (RFT). Elektroneuromyography (ENMG) was performed on all participants with ALS as the gold standard for confirming the diagnosis [18]. Only 10 patients in the control group underwent the procedure due to its painful nature. In further stages of the study, we utilized ABG and RFT reference values published in the literature, corresponding to the age and gender of individuals in the control group. All participants in the control group were over 18 years old, with no signs and symptoms of other neurodegenerative diseases, polyneuropathies, neuromuscular junction disorders, endocrine disorders, oncological diseases, or other concomitant diseases that could affect respiratory function, as well as those taking medications that could affect respiration.

### 2.2. The Phrenic Nerve Examination

**Figure 1:**
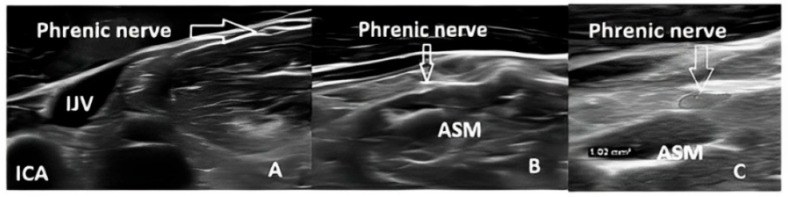
(**A**) Normal phrenic nerve ultrasound in a healthy volunteer. (**B**) Phrenic nerve ultrasound in a participant with ALS. (**C**) Methodology for measuring the cross-sectional area of the phrenic nerve. ASM—anterior scalene muscle; IJV—internal jugular vein; ICA—internal carotid artery.

The phrenic nerve ultrasound was conducted by two authors, both of whom are neurologists experienced in carotid artery and peripheral nerve sonography, using a “Philips EPIQ 7” machine (Arbor Medical Corporation LT, Baltu pr. 145, Kaunas, LT-47125, Lithuani), Kaunas, Lithuania, Arbor Mediacal Corporation LT, with a 4–18 MHz transducer (CE 0086). We used the same nerve ultrasound examination technique as in our previous study [1]. The transducer was placed, and a spindle-shaped hypoechoic structure with a more hyperechoic periphery was identified as the PN on the anterior scalene muscle (Figure 1). Qualitative characteristics (homogeneity, echogenicity) and quantitative measurements (cross-sectional area) were assessed. The area was measured three times by each examiner with a 0.01 mm^2^ margin of error, then averaged. Both examiners were blinded to each other’s results.

### 2.3. Electrophysiological Examination

The PN electrophysiological examination was performed using the Nicolet Natus EDX system, Vilnius, Lithuania, Sormedica (CE 592232). The participants were positioned on their backs, and the skin of the examination area was cleaned with alcohol. The surface and recording electrodes (G1, G2) were coated with gel to improve the contact between the electrodes and the skin. The first electrode (G1) was fixed 5 cm above the sternal angle, and the second electrode (G2) was placed 16 cm from the first electrode along the anterior axillary line. Bipolar stimulation was used for stimulation, where the PN was stimulated between the clavicular head of the sternocleidomastoid muscle and the sternal head in the supraclavicular fossa. Electrical stimulation was performed with 0.1 ms duration pulses [27]. The motor response (M response) was recorded. Measurements were performed during normal inhalation and exhalation, and data on the conduction of the left and right PN were obtained—for analysis, the larger response was used, and the amplitude (ms) and area (mVms) of the motor response were evaluated. Due to the painful nature of the investigation, only 10 volunteers from the control group were able to participate.

### 2.4. Examination of Respiratory Function and Analysis of Arterial Blood Gas

To evaluate respiratory function, patients with ALS were referred for a consultation with a pulmonologist. All participants were evaluated after the diagnosis of ALS was confirmed. During the visit, all participants underwent a chest organ X-ray examination, arterial blood gas analysis (ABG), spirometry (Ganshorn Medizin Electronic, Niederlauer, Germany), and respiratory muscle strength testing (Ganshorn Medizin Electronic, Niederlauer, Germany). The chest X-ray was used to evaluate lung structure, while ABG analysis assessed arterial blood gas parameters, including acid-base balance (pH), partial oxygen pressure (pO_2_), partial carbon dioxide pressure (pCO_2_), base excess/deficit (SBE), bicarbonate concentration (HCO_3_ˉ), and oxygen saturation (SO_2_). Forced vital capacity (FVC) and forced expiratory volume in one second (FEV1) were obtained during spirometry. This test was interpreted according to the American Thoracic Society’s (ATS) recommendations. Respiratory muscle strength was assessed for maximal inspiration (PI max) and expiration (PE max) according to international assessment recommendations [28]. PI max reflects the strength of the diaphragm and internal intercostal muscles, while PE max indicates the strength of the transverse thoracic, external intercostal, and abdominal muscles. The results were evaluated, and the pulmonologist provided a conclusion on respiratory function. Moreover, following confirmation of ALS diagnosis, as pulmonary function, we evaluated arterial blood gas parameters, including pH, pCO_2_, pO_2_, HCO_3_, BE(b), and sO_2_. Control group participants were not included, as there are established international laboratory ranges for these parameters, eliminating the necessity for control comparisons.

### 2.5. Statistical Analysis

The data analysis in our previous publications included the application of both descriptive and comparative statistical analysis methods, utilizing Microsoft Excel and IBM SPSS v29. Normally distributed variables were compared using the two-sided t-test, with means and standard deviations reported. Non-normally distributed variables were analyzed using the Mann–Whitney U test, with median values along with minimum and maximum values provided for group comparison. Statistical hypotheses were tested, and significant differences and dependencies were identified at a significance level of *p* < 0.05.

## 3. Results:

### 3.1. Demographic and Clinic Data

In a prospective study, 32 patients with ALS and 64 control group participants were included. Only newly diagnosed patients with ALS were included and subsequently classified into one of three ALS subgroups, as shown in Table 1. In our comparative analysis between patients with ALS and the control group, no significant differences were found in age or sex distribution. The age of disease onset in the ALS group was 57.97 ± 9.22 years, and the disease duration was 15.41 ± 9.04 months. Although the ALS group demonstrated a lower average height compared to controls (*p* = 0.009), the difference in weight was not statistically significant (*p* = 0.846). Notably, patients with ALS exhibited a significantly smaller hip circumference (*p* = 0.002) and a non-significant reduction in waist circumference (*p* = 0.113). Furthermore, patients with ALS had a significantly higher BMI (*p* = 0.001) compared to controls. Data regarding the demographic and clinical characteristics of the subjects are shown in Table 1.

### 3.2. Phrenic Nerve Sonography Findings in Patients with ALS and Control Group

In the control group, the average cross-sectional area of the phrenic nerve was 1.12 ± 0.02 mm^2^ on the right side and 1.08 ± 0.016 mm^2^ on the left side. In contrast, in patients with ALS, there was a significant decrease in the cross-sectional area of the PN, measuring 0.81 ± 0.02 mm^2^ on the right side and 0.82 ± 0.02 mm^2^ on the left side, with *p*-values less than 0.01 for both sides compared to the control group.

Changes in the homogeneity and echogenicity of the PN were also observed on both sides. On the right side, 43.8% of the nerves showed heterogeneity, 40.6% were isoechoic, and 21.9% were hyperechoic. On the left side, 59.4% of the nerves exhibited heterogeneity, 34.4% were isoechoic, and 28.1% were hyperechoic. More details are shown in Table 2.

Homogeneity on both sides of the phrenic nerve suggests that most cases are either normal or exhibit heterogeneity. Changes observed in the lower motor neuron (LMN) form of ALS indicate that most alterations are prevalent in LMN cases. Similarly, changes in echogenicity, comparable to homogeneity, are observed in LMN and also in the bulbar form on the left side. Moreover, the right side displays a significantly higher COMPASS-31 score (*p* < 0.001), and both sides show significant changes in the ALSFRS-R score (*p* < 0.001). These findings are shown in Table 3.

### 3.3. Phrenic Nerve Changes Findings Associations with Electroneuromyography Examination

The data comparing electrophysiological parameters between the control and ALS groups showed no significant differences in nerve conduction velocity, as indicated by similar latency times for both groups (*p* = 0.498 for the right side and *p* = 0.369 for the left side). However, significant reductions in amplitude and area under the curve are noted in the ALS group on both sides (*p* < 0.001 for all). These findings are shown in Table 4.

### 3.4. Correlations of Phrenic Nerve Changes with Respiratory Function in Patients with ALS

In this study, our analysis specifically aimed to explore potential correlations between these arterial blood gas parameters and the cross-sectional area of the phrenic nerve, as well as to assess any associations with changes in echogenicity and homogeneity. Our findings indicated that there were no significant correlations between the measured blood gas parameters and the anatomical and morphological characteristics of the phrenic nerve.

Respiratory parameters, such as FEV1 (L and %), FVC (L and %), FEV1/FVC ratio, and FEV1/FVC percentage, were also assessed. Our analysis did not reveal any statistically significant correlations between the cross-sectional area of the phrenic nerve and these respiratory parameters. Furthermore, comparisons between conducted study results and respiratory function also showed no significant changes (see Table 5).

## 4. Discussion

The present study offers a comprehensive evaluation of phrenic nerve (PN) sonographic characteristics and their correlations with clinical neurophysiological parameters in patients with amyotrophic lateral sclerosis (ALS). This is the first investigation of its kind conducted in Lithuania, providing valuable insights into the utility of PN ultrasound as a diagnostic and prognostic tool in ALS.

Patients with ALS showed a significantly reduced hip circumference (*p* = 0.002) and a non-significant decrease in waist circumference (*p* = 0.113), suggesting potential muscle loss or weight reduction typical in ALS progression. Furthermore, ALS patients had a significantly lower body mass index (BMI) (*p* = 0.001) compared to controls, which may reflect varying degrees of muscle wasting or differential fat distribution as the disease progresses. Moreover, this condition is associated with an increased risk of death [29,30].

Our findings indicate that the cross-sectional area of the PN is significantly reduced in patients with ALS compared to the control group. This observation aligns with previous studies that have demonstrated a reduction in nerve size associated with motor neuron degeneration in ALS [24,31]. The reduction in PN size likely reflects the atrophy of motor neurons and muscle fibers, aligning with the pathophysiological mechanisms underlying ALS. It is supported by post-mortem studies [32], which revealed a loss of large, myelinated axons, predominantly in distal regions, as well as significant distal axonal PN atrophy. One of the pathogenic mechanisms involves oxidative damage leading to axonal dysfunction and degradation [33]. Oxidative stress, influencing diffuse cellular processes, may explain the diffuse degeneration of both the right and left PN. Additionally, we evaluated echogenicity and homogeneity changes. Heterogeneous and hyperechoic changes in the PN were observed more frequently in the ALS group, suggesting structural and compositional alterations within the nerves. These sonographic changes are likely due to the degenerative processes affecting nerve fibers and surrounding tissues, supporting the potential of PN ultrasound as a non-invasive biomarker for ALS, as discussed in our recent study [1]. The assessment of homogeneity and echogenicity revealed significant alterations in patients with ALS; however, there are no existing studies available for direct comparison.

Despite observing clear morphological differences in PN sonography, our study did not establish significant correlations between PN size and parameters of arterial blood gas analysis or various respiratory function parameters. These findings contrast with those of Heiman-Patterson et al. (2021), who reported that pulmonary function decline is associated with a worse prognosis for ALS [34]. This discrepancy suggests that while PN ultrasound can provide detailed structural insights, it may not directly reflect the functional respiratory impairment or systemic biochemical changes in patients with ALS. This highlights the complexity of ALS pathology, where multiple factors contribute to disease progression and clinical manifestations. It is important to emphasize that the results of spirometry and respiratory muscle strength tests depend on the patient’s effort and, in some cases, the form or severity of the disease. In contrast, PN electrophysiological testing requires less patient involvement.

Electroneuromyography (ENMG) data also revealed significant differences in amplitude and area between patients with ALS and controls, consistent with findings from other studies that reported amplitude and latency changes in PN conduction studies [26]. These results align with the established role of ENMG in diagnosing ALS by providing direct evidence of motor neuron dysfunction. Some authors suggest that ENMG is more sensitive in assessing respiratory distress in ALS compared to phrenic nerve CSA [20,35]. However, the painful nature of ENMG limited its application in participants, highlighting the need for less invasive diagnostic methods, such as ultrasound.

Interestingly, the demographic analysis revealed no significant differences in age or sex distribution between patients with ALS and controls, although patients with ALS exhibited a lower BMI. This lower BMI could be reflective of reduced physical activity due to muscle weakness and paralysis, which is common in ALS. The observed demographic characteristics are consistent with the known epidemiology of ALS, which predominantly affects older adults with a slight male predominance.

The analysis revealed significant correlations between phrenic nerve ultrasound findings and clinical scores, particularly for Compass-31 and the ALS functional rating scale-revised (ALSFRS-R). On the right side, Compass-31 showed strong statistical significance (*p* < 0.001), suggesting that autonomic dysfunction, as assessed by Compass-31, may be associated with changes in phrenic nerve morphology. However, on the left side, Compass-31 did not show a significant correlation (*p* = 0.089).

For ALSFRS-R, significant correlations were observed on both sides, with *p* < 0.001 for both the right and left phrenic nerve ultrasound assessments. This suggests that phrenic nerve alterations are closely linked to the functional impairments assessed by ALSFRS-R, which reflects the overall severity of ALS-related motor dysfunction. Our study has several strengths, including a well-defined cohort, rigorous sonographic evaluation, and a comprehensive analysis of multiple parameters. However, it also has limitations, such as the relatively small sample size and the exclusion of certain control participants from ENMG due to discomfort associated with the procedure. Future studies should consider increasing the number of patients with ALS to further validate these findings and explore longitudinal changes in phrenic nerve (PN) ultrasound characteristics, as well as the potential variability in nerve changes across different age groups and stages of the disease. Longitudinal studies could also help track the progression of phrenic nerve degeneration and its relationship to respiratory dysfunction in ALS. Future research should aim to include larger cohorts to further validate the utility of PN ultrasound in ALS diagnosis and prognosis.

## 5. Conclusions

Our study highlights the importance of phrenic nerve ultrasound as a promising supplementary diagnostic tool for ALS. The significant differences in phrenic nerve size, echogenicity, and homogeneity between patients with ALS and the control group demonstrate that ultrasound imaging can detect morphological changes in the phrenic nerve. Incorporating phrenic nerve ultrasound into routine diagnostic protocols could improve early detection, enhance disease monitoring, and offer a more comprehensive understanding of the neurodegenerative processes in ALS. This tool could be particularly valuable in cases where ENMG results are inconclusive, making it a useful complement to existing diagnostic methods. Further research and larger-scale studies are needed to confirm these findings and explore the full potential of phrenic nerve ultrasound in clinical practice for ALS diagnosis and management.

## Figures and Tables

**Table 1:** Demographic and clinical characteristics of patients with ALS and control group.

Variable	Control Group	ALS Group	*p* Value ^a^
Number	64	32	
		LMN—14 UMN—9 Bulbar—9	
Age years, mean ± SD (range, years)	60.84 ± 10.67	59.34 ± 9.93	*p* = 0.508
Sex (M:F)	1:1.37	1.28:1	*p* = 0.193 ^b^
Weight kg, mean ± SD (range, kg)	80.81 ± 14.48	72.22 ± 15.59	*p* = 0.846
Height cm, mean ± SD (range, cm)	170.56 ± 9.92	170.16 ± 8.98	* p * = 0.009
Hip circumference ± SD (range, cm)	103.19 ± 8.07	98.97 ± 13.62	* p * = 0.002
Waist circumference ± SD (range, cm)	92.92 ± 10.48	84.84 ± 13.87	*p* = 0.113
BMI Kg/m^2^ ± min/max (range, Kg/m^2^)	27.72 (18.67–36.63)	24.89 (18.99–36.57)	* p * = 0.001 ^c^

^a^ Student’s *t*-test; ^b^ Χ^2^ test for equality of proportions; ^c^ Mann–Whitney U test; BMI—body mass index. ALS—amyotrophic lateral sclerosis; LMN—lower motor neuron; UMN—upper motor neuron.

**Table 2:** Phrenic nerve echogenicity and homogeneity changes in patients with ALS and control group.

		Control Group Right	ALS Right	*p* Value	Control Group Left	ALS Left	*p* Value
Homogeneity	Homogenous	92.2%	43.8%	**<0.001**	87.5%	40.6%	**<0.001**
	Heterogenous	7.8%	56.2%	**<0.001**	12.5%	59.4%	**<0.001**
Echogenicity	Hypoechoic	75%	37.5%	**<0.001**	78.1%	37.5%	**<0.001**
	Isoechoic	25%	40.6%	**<0.001**	21.9%	34.4%	**<0.001**
	Hyperechoic	0%	21.9%	**<0.001**	0%	28.1%	**<0.001**

Χ2 test for equality of proportions. Ultrasonic features of the PN nerve in the group of patients diagnosed with amyotrophic lateral sclerosis (ALS) (*n* = 32), showing homogeneity and echogenicity. Abbreviations: ALS—amyotrophic lateral sclerosis; %—percentage.

**Table 3:** Phrenic nerve changes associations with demographic, clinic and ultrasound findings in ALS.

	Right PN US Patients Nr.—32	Left PN US Patients Nr.—32
Demographics		
**Height cm**	***p*-0.269**	***p*-0.124**
**Weight kg**	***p* < 0.016**	***p* < 0.001**
**BMI kg/m^2^**	***p* < 0.026**	***p*-0.004**
**Waist circumference cm**	***p* < 0.025**	***p*-0.011**
**Hip circumference cm**	*p*-0.149	*p*-0.053
**Sex**	*p*-0.777	*p*-0.974
**Age**	*p*-0.894	*p*-0.348
**Age at disease onset**	*p*-0.728	*p*-0.34
**Duration of illness**	*p*-0.529	*p*-0.704
**Compass-31**	***p* < 0.001**	*p*-0.089
**ALFSR-R**	***p* < 0.001**	***p* < 0.001**

Abbreviations: PN—phrenic nerve; US—ultrasound; BMI—body mass index; Compass-31—composite autonomic symptom score-31 scale. ALFSR-R—the revised amyotrophic lateral sclerosis functional rating scale. Values except gender are mean SD. Significance of bold values: *p* < 0.05.

**Table 4:** ENMG between ALS and control group.

	Control Group Patients Nr.—64	ALS Group Patients Nr.—32	*p* Value
**Latency right ms ± min/max (range, ms)**	6.75 (4.5–7.8)	6.40 (2.5–11.9)	0.498
**Amplitude right mV ± min/max (range, mV)**	1.25 (0.9–1.9)	0.40 (0.1–1.0)	**<0.001**
**Area right mVms ± min/max (range, mVms)**	11.20 (5.4–17.3)	3.80 (0.6–10.2)	**<0.001**
**Latency left ms ± min/max (range, ms)**	7.10 (4.8–8.0)	6.65 (2.7–10.1)	0.369
**Amplitude left mV ± min/max (range, mV)**	1.35 (1.0–1.8)	0.4 (0.1–6.0)	**<0.001**
**Area left mVms ± min/max (range, mVms)**	11.15 (6.2–17)	3.85 (0.9–10.1)	**<0.001**

Significance of bold values: *p* < 0.05.

**Table 5:** Correlation between EMG test results and respiratory function.

ALS Patients Nr.—32		FEV1 l	FEV1 %	FVC l	FVC %	FEV1/FVC	FEV1/FVC %
**Latency**	**Right**	0.05	0.18	0.13	0.09	0.11	0.12
	**Left**	0.09	0.22	0.21	0.15	0.33	0.37
**Amplitude**	**Right**	0.77	0.13	0.23	0.14	0.62	0.74
	**Left**	1.00	0.17	0.65	0.14	0.86	0.86
**Area left**	**Right**	0.94	0.19	0.48	0.16	0.93	0.84
	**Left**	0.73	0.15	0.33	0.16	0.79	0.91

Significance values: *p* < 0.05.

## Data Availability

The original contributions presented in the study are included in the article, further inquiries can be directed to the corresponding authors.
